# Transcriptomic profiling of Gh/Igf system reveals a prompted tissue-specific differentiation and novel hypoxia responsive genes in gilthead sea bream

**DOI:** 10.1038/s41598-021-95408-6

**Published:** 2021-08-12

**Authors:** F. Naya-Català, P. Simó-Mirabet, J. Calduch-Giner, J. Pérez-Sánchez

**Affiliations:** Nutrigenomics and Fish Growth Endocrinology, Institute of Aquaculture Torre de La Sal, IATS-CSIC, 12595 Castellón, Spain

**Keywords:** Physiology, Transcriptomics

## Abstract

A customized PCR-array was used for the simultaneous gene expression of the Gh/Igf system and related markers of muscle growth, and lipid and energy metabolism during early life stages of gilthead sea bream (60–127 days posthatching). Also, transcriptional reprogramming by mild hypoxia was assessed in fingerling fish with different history trajectories on O_2_ availability during the same time window. In normoxic fish, the expression of almost all the genes in the array varied over time with a prompted liver and muscle tissue-specific differentiation, which also revealed temporal changes in the relative expression of markers of the full gilthead sea bream repertoire of Gh receptors, Igfs and *Igf*-binding proteins. Results supported a different contribution through development of *ghr* and *igf* subtypes on the type of action of GH via systemic or direct effects at the local tissue level. This was extensive to Igfbp1/2/4 and Igfbp3/5/6 clades that clearly evolved through development as hepatic and muscle Igfbp subtypes, respectively. This trade-off is however very plastic to cope changes in the environment, and *ghr1* and *igfbp1/3/4/5* emerged as hypoxic imprinting genes during critical early developmental windows leading to recognize individuals with different history trajectories of oxygen availability and metabolic capabilities later in life.

## Introduction

The link between a poor early life environment and increased risk for growth and metabolism disorders has been evidenced in human epidemiological studies, and in a vast array of studies across non-human primates, pigs, sheep and rodents^1,2^. In particular, poor maternal nutrition, including undernutrition and overnutrition, leads to obesity and cardiovascular disorders with an offspring phenotype that closely resembles that of growth hormone (GH) deficiency^3,4^. Initially, developmental programming was considered an irreversible change in developmental trajectory, but a number of experimental studies evidence that this process is reversible during critical early windows of developmental plasticity. Thus, intervention strategies using GH or its downstream regulators ameliorate most functional disorders arising from defects in developmental programming^5,6^. Specifically, adult-onset obesity seen in pups born to undernourished rat dams can be prevented with pre-weaning GH treatment^7^. Pre-weaning GH treatments of undernourished offspring also prevents the appearance of a pro-inflammatory phenotype later in life^8^. Otherwise, fetal programming is a consequence of hypoxia, and the transcription factor HIF-1 coordinates the chromatin remodeling of a wide range of target genes including those of the insulin-like growth factor (IGF) system^9,10^. Experimental evidence also supports the participation of epigenetic mechanisms on the developmental programming of GH/IGF axis. Thus, differential methylation of IGF-I and its binding proteins is likely to play a role in the pathogenesis of small gestational age of neonates^11^. Similarly, in rats, maternal low protein dietary intake alters the offspring hepatic IGF2 expression via alterations in its DNA methylation pattern^12,13^. However, most of these studies remain largely associative in nature with limited evidence for direct causality, transgenerational effects or sexually dimorphic responses to therapeutic treatments^6^.

In livestock fish, special emphasis on epigenetic regulation has been focused on the nutritional programming of lipid metabolism^14–16^, and differential promoter DNA methylation supports adaptive offspring changes in the gene expression of the delta 9 fatty acid desaturase (*scd1a*) in gilthead sea bream (*Sparus aurata*) fed plant-based diets^17^. Also, as reviewed by Jonsson and Jonsson^18^, there is now evidence that the offspring performance of fish is affected by the temperature during embryo development or by the temperature experienced by parents prior to egg fertilization^19,20^. Developmental temperature had also persistent effects on the muscle growth responses experienced by gilthead sea bream juveniles^21^, and overall these findings evidence that myogenesis through developmental growth is modulated by the temperature during early life stages.

Oxygen (O_2_) availability is another main factor that affects the offspring performance of fish. Certainly, severe hypoxia during embryo and larval development in zebrafish has served to identify novel hypoxia-protective genes^22^, though it appears that the induction of *igfbp1* expression is a conserved physiological mechanism to restrict the Igf-stimulated growth and developmental process under hypoxic stress^23^. Otherwise, studies in this model fish species have demonstrated that parental hypoxic exposure improves the offspring hypoxia resistance^24^. Conversely, hypoxia preconditioning of adults of *Oryzias melastigma* causes transgenerational reproductive impairments that persisted in F1 and F2 generations^25^. As part of this complex cross-talk between O_2_ levels and metabolic rates, the induction of a hypometabolic state is one of the clearest responses of individuals facing severe or mild hypoxia episodes. Indeed, the maintenance of aerobic metabolism is recognized as a primary hypoxia survival strategy in most organisms, including fish^26^. Specifically, in gilthead sea bream, mitochondrial bioenergetics of blood cells are finely adjusted at the transcriptional level by changes in water O_2_ concentrations, contributing the enhanced expression of the last electron acceptor of the mitochondrial respiratory chain (Complex IV) to ensure an efficient aerobic ATP production under severe hypoxia^27^. In the same fish species, tissue expression patterns of genes on the category of energy metabolism and *Gh/Igf* system also contribute to differentiate different O_2_ status and rearing densities in a 3-weeks mild hypoxia trial of juvenile fish^28^. Moreover, mild hypoxia preconditioning improves swimming performance, being supported this metabolic feature by blood biochemistry and muscle transcriptional profiling of exercised fish^29^.

Also in gilthead sea bream, mild hypoxia imprinting during early stages (60–80 days post-hatching, dph) increases hypoxia resilience in fish facing a second hypoxia episode (112–127 dph)^30^. The present study aimed to depict these hypoxic effects upon the Gh/Igf system in conjunction with markers of muscle growth and energy/lipid metabolism, with the objective to provide new insights in early hypoxic and developmental Gh/Igf programming. Such approach contributes to solve the gaps of knowledge arising from the recently reviewed nutritional and environmental regulation of Gh/Igf axis across the production cycle of gilthead sea bream^31^, a highly cultured fish in all the Mediterranean basin. This might also serve to further disclose the use of downstream regulators of Gh as surrogate markers of fish welfare and fingerlings quality.

## Results

### The targeted gene approach depicts a developmentally regulated gene expression profile

Fish were sampled during the 60–127 days post-hatching (dph) window to assess developmentally mediated changes in the gene expression profile of whole-larva (60 dph), and specific tissues (skeletal muscle, liver) of fish fingerlings (81/112/127 dph). This was a targeted transcriptomic approach based on the use of a customized PCR-array for the simultaneous gene expression profiling of selected markers of Gh/Igf system, muscle cell growth and differentiation, and lipid/energy metabolism (Table 1). Such approach yielded 26 genes out of 28 that varied significantly (one-way ANOVA, *P* < 0.05) over time when comparisons are made between whole-larva and skeletal muscle through all the experimental period (Table 2, Supplemental Table 1). In liver, the number of differentially expressed genes was reduced to 19 through the analyzed 81–127 dph window, with a large representation of markers of Gh/Igf system and lipid /energy metabolism within the set of differentially expressed genes (Table 2, Supplemental Table 2).

**Table 1:** PCR-array layout for gene expression profiling.

Function	Gene	Symbol	GeneBank
GH/IGF system	Growth hormone receptor-type 1	*ghr1*	AF438176
	Growth hormone receptor-type 2	*ghr2*	AY573601
	Insulin-like growth factor 1	*igf1*	AY996779
	Insulin-like growth factor 2	*igf2*	AY996778
	Insulin-like growth factor binding protein 1a	*igfbp1a*	KM522771
	Insulin-like growth factor binding protein 1b	*igfbp1b*	MH577189
	Insulin-like growth factor binding protein 2a	*igfbp2a*	MH577190
	Insulin-like growth factor binding protein 2b	*igfbp2b*	AF377998
	Insulin-like growth factor binding protein 3a	*igfbp3a*	MH577191
	Insulin-like growth factor binding protein 3b	*igfbp3b*	MH577192
	Insulin-like growth factor binding protein 4	*igfbp4*	KM658998
	Insulin-like growth factor binding protein 5a	*igfbp5a*	MH577193
	Insulin-like growth factor binding protein 5b	*igfpb5b*	MH577194
	Insulin-like growth factor binding protein 6a	*igfbp6a*	MH577195
	Insulin-like growth factor binding protein 6b	*igfbp6b*	MH577196
	Insulin receptor	*insr*	KM522774
	Insulin-like growth factor 1a receptor	*igf1ra*	KJ591052
	Insulin-like growth factor 2 receptor	*igf2r*	KM522776
Muscle cell proliferation and differentiation	Myogenic factor MYOD2	*myod2*	AF478569
	Myostatin/Growth differentiation factor 8	*mstn/gdf8*	AF258448
	Myocyte-specific enhancer factor 2A	*mef2a*	KM522777
	Myocyte-specific enhancer factor 2C	*mef2c*	KM522778
	Follistatin	*fst*	AY544167
Lipid and energy metabolism	Citrate synthase	*cs*	JX975229
	Carnitine palmitoyltransferase 1a	*cpt1a*	JQ308822
	Proliferator-activated receptor gamma coactivator 1 alpha	*pgc1α*	JX975264
	Sirtuin 1	*sirt1*	KF018666
	Sirtuin 2	*sirt2*	KF018667

**Table 2:**
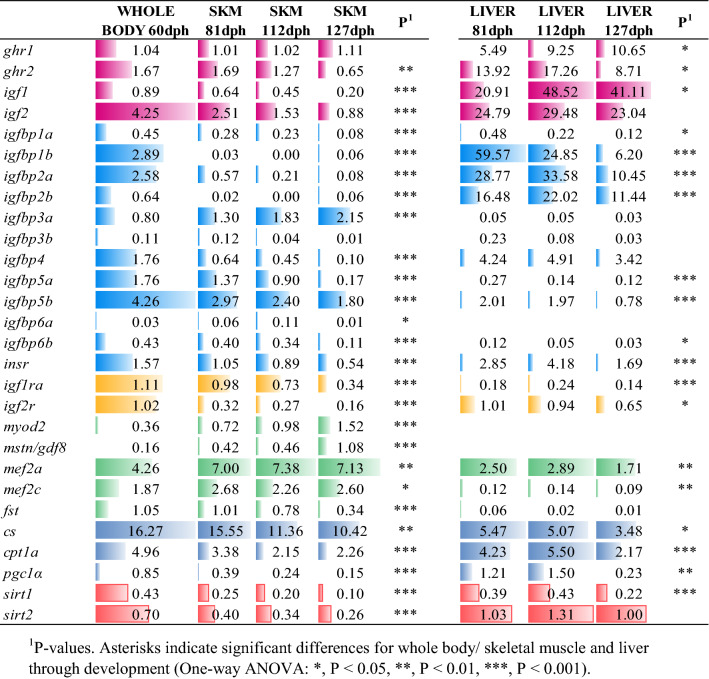
Relative gene expression profiling of whole body, skeletal muscle and liver of gilthead sea bream during early stages (60–127 dph).

Data are the mean of 6 fish. Values of skeletal muscle were in reference to the expression level of *igf2r* of whole body with an arbitrary assigned value of 1. Values of liver were in reference to the expression level of *igf2r* in fish sampled at 81dph with an arbitrary assigned value of 1. Bar colors represent the magnitude of change within a given gene category: *ghr/igf* (pink), *igfbp* (light blue), *insr/igfr* (yellow), muscle cell proliferation and differentiation factors (green), oxidative markers (dark blue) and energy sensors (red).

^1^*P*-values. Asterisks indicate significant differences for whole body/ skeletal muscle and liver through development (One-way ANOVA: *P < 0.05, **P < 0.01, ***P < 0.001).

### The repressed activity of muscle Gh/Igf system is concurrent with myoblast cell differentiation

At a closer look, targeted genes of skeletal muscle were progressively up- or down-regulated over time, taken whole-larva with a high contribution of skeletal muscle as the initial reference time (Table 2). Thus, the expression of *gh receptor-type 2* (*ghr2*) was maximal in whole-larva, decreasing thereafter until the achievement of the lowest expression level at 127 dph. Meanwhile, the expression of *gh receptor-type 1* (*ghr1*) remained unchanged. In parallel, the muscle expression of *igf1* and *igf2* was decreased over time, remaining the expression of *igf2* greater than *igf1*. With the exception of *igfbp3a,* the expression of *igf* binding proteins was higher in whole-larva that in the skeletal muscle of developing fish fingerlings. Thus, up to 6 *igfbp* subtypes (*igfbp1a*, igfbp1b, *igfbp2a, igfbp2b, igfbp3b, igfbp6a*) were expressed at very low levels in the skeletal muscle of fish sampled at the end of trial. The muscle expression of *igfbp5b* and *igfbp6b* also decreased over time, though that of *igfbp5b* remained at a relative high expression level at 127 dph. Noteworthy, the muscle expression of insulin receptor (*insr*) and *igf* receptors (*igf1ra*, *igf2r*) was also progressively decreased through development. In contrast, the expression of myogenic factors (myostatin, *mstn*; myoblast determination protein 2, *myod2*; myocyte-specific enhancer protein 2a, *mef2a*, myocyte-specific enhancer protein 2c, *mef2c*) driving muscle cell differentiation were overexpressed over time. The opposite was found for follistatin (*fst*), a well-known activin antagonist of *mstn.* Lastly, all the analyzed markers of lipid and energy metabolism (citrate synthase, *cs*; carnitine palmitoyltransferase 1a, *cpt1a*; proliferator-activated receptor gamma coactivator 1 alpha, *pgc1α*; sirtuin 1, *sirt1*; sirtuin 2, *sirt2*) were significantly down-regulated over time.

### Developmental changes in the hepatic Gh/Igf system are first elicited by the enhanced expression of *ghr1*

As also shown in Table 2, the developmental expression pattern of hepatic *ghr1* and *ghr2* was opposite, with a two-fold increase in *ghr1* expression that was concurrent with a suppressed *ghr2* expression during the 81–127 dph window. Noteworthy, the up-regulated expression of *igf1* would mirror changes in *ghr1*, whereas *igf2* expression remained unchanged. Regarding *igfbps*, the general pattern was a down-regulated expression over time, which was statistically significant for *igfbp1a*, *igfbp1b, igfbp2a*, *igfbp2b, igfbp5a, igfbp5b* and *igfbp6b* regardless of differences in relative gene expression. At a lowest extent, this was extensive to *igfbp4* and *igfbp6a*. The developmental trend for *insr, igf1ar* and *igfr2* was also a down-regulated gene expression with the lowest expression level for *igf1ra*. This expression pattern was also extensive to *mef2a*, whereas mRNA transcripts of *fst* and other myogenic factors (*myod2, mstn, mef2c*) remained almost undetectable all the time. Lastly, with the exception of *sirt2,* markers of lipid and energy metabolism were down-regulated over time, reflecting a time-dependent decrease on energy demand with the progression of fish development.

### The early window is becoming a sensitive period for hypoxia preconditioning

**Figure 1:**
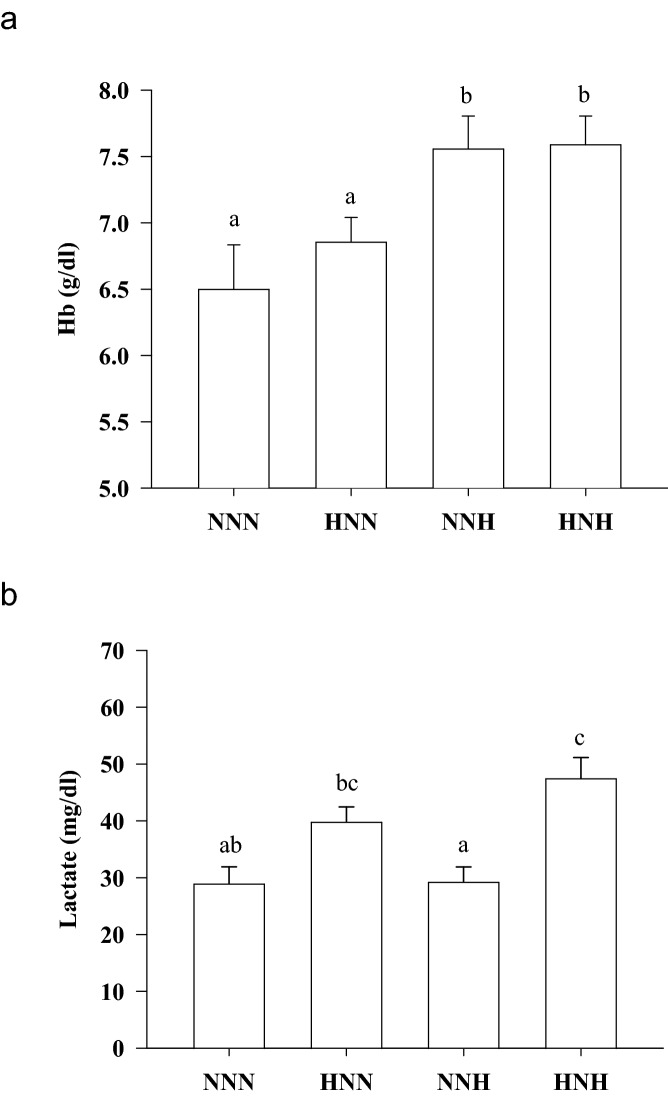
Blood haemoglobin (**a**) and lactate (**b**) levels of normoxic fish (NNN), early hypoxic fish (HNN), late hypoxic fish (NNH), and early and late hypoxic fish (HNH). Values are the mean ± SEM of 10–12 fish. Different letters indicate statistically significant differences among groups (Student Newman–Keuls test, *P* < 0.05).

**Figure 2:**
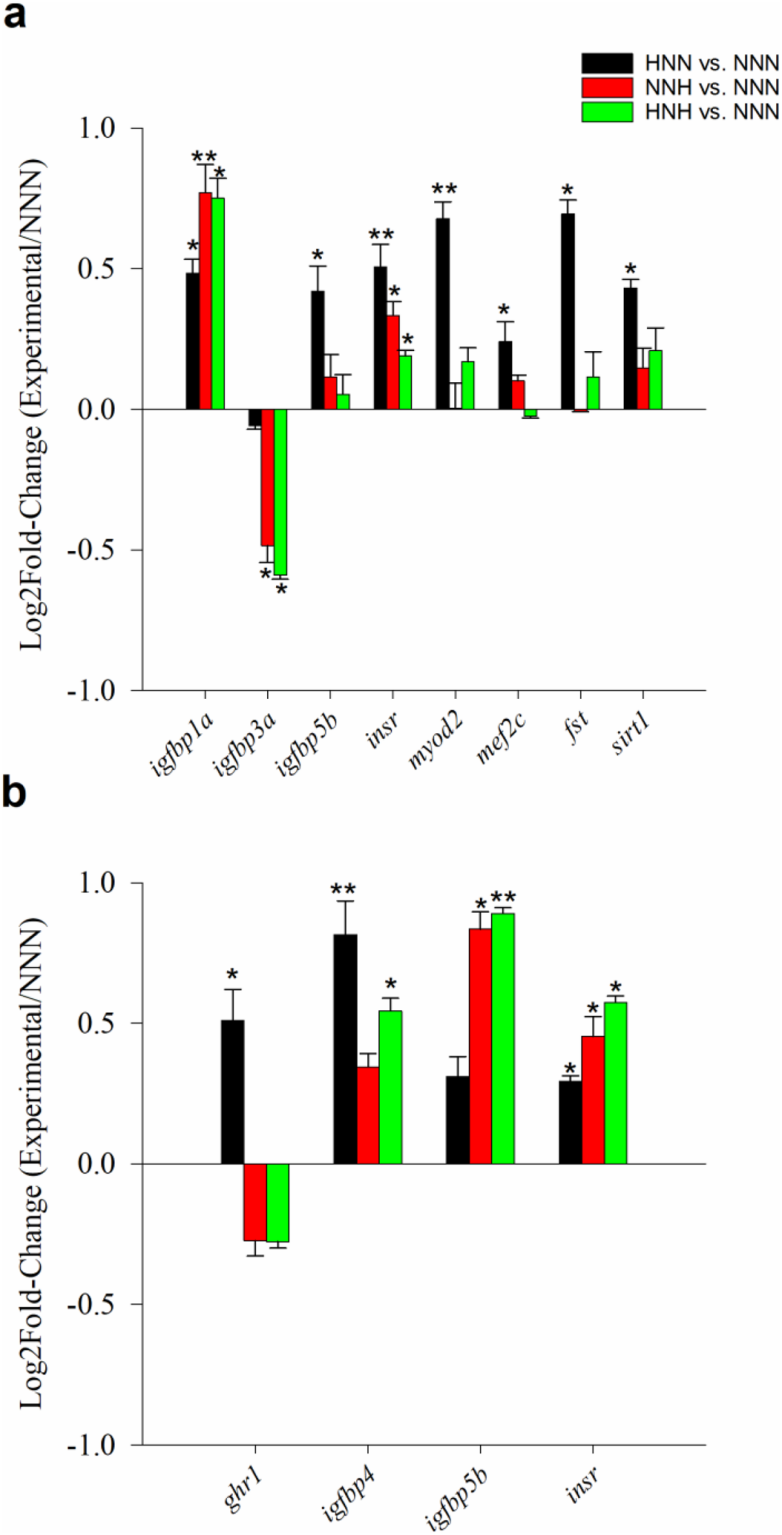
Fold-change (relative to normoxic fish, NNN) of differentially expressed genes in muscle (**a**) and liver (**b**) of gilthead sea bream fingerlings (127 dph) with different history trajectories of O_2_ availability: early hypoxic (HNN, black bars), late hypoxic (NNH, red bars), and early and late hypoxic (HNH, green bars). Values are the mean ± SEM of 6 fish. Asterisks indicate statistically significant differences with NNN group (*t* test, *P < 0.05; **P < 0.01).

The effects of different trajectories in O_2_ availability were assessed at 127 dph. This rendered 4 experimental groups, according to the timing and number of hypoxia stimuli through the analyzed 60–127 dph window (normoxic fish, NNN; early hypoxic fish, HNN; late hypoxic fish, NNH; early and late hypoxic fish, HNH). The physiological success of changes in O_2_ availability was first evaluated by measures of circulating levels of hemoglobin (Hb) that evidenced an enhanced O_2_ transport capacity in late hypoxic fish (NNH and HNH) (Fig. 1a). However, plasma lactate levels primarily reflected the early hypoxia exposure, being increased the circulating levels of this downstream marker of anaerobic metabolism in HNN and HNH fish, but not in fish facing for the first time a late hypoxic stimulus (NNH fish) (Fig. 1b). At the transcriptional level, up to 8 genes were differentially expressed in response to mild hypoxia exposure in the skeletal muscle of fish sampled at 127 dph, but again different patterns of response were found (Fig. 2a, Supplemental Table 3). First, muscle *igfbp1a* expression was induced by early or late hypoxia with the highest amount of *igfbp1a* transcripts in NNH and HNH fish. By contrast, *igfbp3a* was apparently refractory to early hypoxia, whereas late hypoxia was effective in down-regulating *igfbp3a* transcripts in both NNH and HNH fish. Lastly, *igfbp5b*, *insr*, *myod2*, *mef2c*, *fst* and *sirt1* were up-regulated by early hypoxia with a maximum expression level in HNN fish, and a recovery of control values in NNH and HNH fish. In liver, the impact of hypoxia intervention yielded four differentially expressed genes corresponding to two main patterns of response that serve to highlight early hypoxia as a more sensitive window for metabolic programming later on (Fig. 2b, Supplemental Table 4). According to this, the expression of *ghr1* and *igfbp4* was up-regulated in HNN fish with a total or partial recovery of control values in NNH and HNH fish. Conversely, the expression of *igfbp5* and *insr* was up-regulated over time by both early and late hypoxia with high expression values in fish exposed to single (HNN, NNH) or double (HNH) mild hypoxia episodes.

## Discussion

Large scale analysis of gene expression of Yúfera et al.^32^ highlighted a high number of genes with circadian variations in gilthead sea bream larvae in comparison to changes found through fish ontogeny^33^ or in response to nutritional programming interventions^34^. Nevertheless, we found in the present work that almost all the genes included in the PCR-array varied significantly over the study, covering the transition from late larval stages to developed fish fingerlings. This may be the result of the over-representation in the PCR-array of genes with a key role in the transition of daily specific growth rates from 10 to 5% over a relatively short period of time^30^, contributing this study to solve some of the gaps on the regulation of the Gh/Igf system in fish. To the best of our knowledge, this is the first study analyzing at a closer look in a model fish farm the full Gh/Igf system repertoire in response to early life development and hypoxia preconditioning.

### Duplicated Ghrs are differentially regulated in a temporal and tissue-specific basis

Large body of evidence demonstrates the differential and tissue-specific regulation of *ghr* subtypes by nutrition and season in juveniles and adults of gilthead sea bream (reviewed in^31^). Thus, in this fish species, hepatic transcripts of *ghr1* (in a low extent *ghr2*) mirror changes in growth rates, circulating levels of Igf1 and hepatic *igf1* transcripts, indicating a prominent role of Ghr1 rather than Ghr2 in the systemic and endocrine growth-promotion action of Gh via hepatic Igfs. Thus, deceleration of growth in gilthead sea bream juveniles fed plant-based diets or semi-synthetic feeds formulated to be deficient in specific nutrients is concurrent with a down-regulated expression of hepatic *ghr1*, resulting in a pronounced decrease of the *ghr1/ghr2* expression ratio at the liver level. The same trend is often found in skeletal muscle, but this adjustment reflects the overexpression of *ghr2*, which would mediate some kind of compensatory growth through the enhanced expression of *igf2*^31^. Muscle expression of *ghr2* was increased by fasting in trout^35^ and gilthead sea bream^36^. By contrast, also in gilthead sea bream, improved growth in response to sustained exercise down-regulated muscle *ghr*2^37^, whereas crowding stress triggered hepatic *ghr2* expression^38,39^. The differential regulation of fish Ghrs is further supported in the present study both in whole-larva and liver/muscle tissues of fish fingerlings, with Ghr1 becoming the predominant subtype at hepatic level as development advances (81–127 dph), with the concurrent decrease of hepatic *ghr2*. A developmental pattern was less evident in skeletal muscle, though it is well recognized that *ghr2* is the highest expressed receptor in skeletal and cardiac muscle tissues of juveniles and adults of gilthead sea bream^28,40^. Also, as pointed out before (see^41^), one fish Ghr might be more responsive of the transmission of the Gh lipolytic signals, while the other will be more active in transmitting growth-promoting signals. More work is needed to stablish these explicit links, but the divergent developmental pattern further supported a different contribution of each *ghr* subtype on the type and mode of action of GH via systemic or direct effects at the local level. Certainly, the main components of somatotropic axis (Gh, Ghrs, Igfs) are produced as soon as transcription starts in fish embryos^42^, and early binding studies revealed a high concentration of actively transcribed *ghrs* in the head of sea bream larvae few days after hatching, which is consistent with their allometric growth^43^. However, this complex trade-off is changing later in life, becoming a matter of discussion how the varying contribution of systemic and local growth-promoting effects on global growth are indicative of a different performance and welfare of farmed fish along live and production cycles (see below).

### The differential hepatic and muscle expression of igf1 and igf2 is elicited early through development

IGF2 is considered a primary growth factor during early life^44,45^, while IGF1 is required for maximal growth later in life^46^. However, substantial amounts of IGF2 are found in humans and a wide-range of fish including gilthead sea bream. As a general trend, overexpression of *igf2* is especially evident in extrahepatic tissues, ranging the expression quotient of *igf2/igf1* from 0.5 in liver to 3–9 in skeletal muscle and to 75–100 in heart, intestine and gonads of juveniles and adults of gilthead sea bream^31^. These expression quotients are in the range reported herein in fish sampled at 127 dph, which is indicative that the *igf2/igf1* trade-off is achieved early through development in gilthead sea bream, especially in the case of skeletal muscle where the *igf2/igf1* expression quotient remained almost invariant and near to 4 from 60 to 127 dph. However, the hepatic overexpression of *igf1* was delayed over time, and a threshold level of 0.5 for the *igf2/igf1* expression quotient was not achieved until 112 dph. Importantly, both in the present and previous studies, *igf* measures considered the expression of the totality of *igf1* transcripts given the retention of the core mature peptide in all Igf1 precursors^47^.

### *igfbp1/2/4* and* igfbp3/5/6* clades also evolve with a differential expression pattern in liver and muscle tissues

The ancestral *igfbp* gene was duplicated in tandem during an early stage of vertebrate evolution to produce a pair of IGFBPs that gave rise in subsequent genome duplication events the two IGFBP clades of modern vertebrates (IGFBP1/2/4; IGFBP3/5/6)^48,49^. Additionally, the third and fourth round of whole genome duplications generated the corresponding paralog pairs. The resulting number of *Igfgbp* subtypes is thereby variable between fish lineages, and searches in the gilthead sea bream genome database (www.nutrigroup.iats-org/seabreamdb;^50^) have identified 11variants, covering the full *igfbp1* to *6* repertoire with paralogs pairs of *igfbp1, 2, 3, 5* and *6*. The identity of these *igfbp* sequences has been corroborated by phylogenetic analyses, indicating gene expression analysis of adult fish that mRNA transcripts of the Igfgp1/2/4 clade are highly represented in the liver tissue of gilthead sea bream, whereas the Igfbp3/4/5 clade is over-represented in the skeletal muscle. This was based on the expression analysis of *igbp1a, igbp2b, igbp3, igfbp4, igfbp5b* and *igfbp6b*. In the present study, the gene expression profiling was extended to all the known *igfbp* repertoire of gilthead sea bream, which corroborates the differential expression of *igfbp* clades in liver and skeletal muscle from early life stages, representing *igfbp1b, igfbp2a, igbp2b* and *igfbp4* the 97.5–98% of total *igfbp* mRNA transcripts expressed in the liver of fish fingerlings. Conversely, *igfbp3a* and *igfbp5b* represented more than 99% of *igfbp* mRNA transcripts early detected in skeletal muscle at 127 dph. Therefore, this is another example of the different transcriptional regulation of Gh/Igf system in hepatic and extra-hepatic tissues, which is early accomplished through development. However, the dominance of muscle *igfpb3a* upon *igfbp5b*, characteristic of adult fish^31^, was delayed herein until 127 dph.

### Insulin/Igf receptors: evolutionary and development prospect

The early completion of developmental changes in the Gh/Igf system is also extensive to insulin and Igf receptors. Fish are indeed the first group of vertebrates in which there is evidence of distinct insulin and Igf molecules and receptors, though important differences regarding binding receptor specificity and abundance has been reported^51–53^. For instance, unlike bird and mammals, binding studies support a low number of insulin receptors in skeletal and cardiac muscles of fish, amphibians and reptiles. However, transcriptional studies in gilthead sea bream indicate that the amount of mRNA transcripts of insulin receptors is similar or even higher than those reported for *igfr1a* and *igf2r* genes across season^31^. The same pattern was found herein during early development with a lowest expression level for *igf1ra* in liver, and *igf2r* in skeletal muscle.

### Hypoxic imprinting

Hypoxia is a major environmental problem in coastal marine ecosystems, and hepatic *igfbp1* transcripts are emerging as a useful biomarker of environmental hypoxia in Atlantic croaker^54^. *igpb1* is also a hypoxia-inducible gene during embryonic growth in zebrafish, and its knockdown alleviates the hypoxia induced growth retardation and developmental delay^23^. Likewise, we observed herein that both early and late hypoxia induced the muscle expression of *igfpb1a*, reinforcing the growth inhibitory effects of igfbp1 paralogs in fasted, refed and *gh-*transgenic fish^55,56^. Less clear is the role of *igfbp3* paralogs as they exert both growth-promoting or inhibitory roles depending of the physiological context^57,58^. This functional dualism is also extensive to gilthead sea bream when considering the transcriptional regulation of *igfbp3* mRNA transcripts in this and previous studies. For instance, the expression of muscle *igfbp3a* was largely induced with the developmental decrease of growth rates during early life stages, as reported previously for the growth impairments due to phosphorous deficiencies in juvenile fish^31^. Conversely, we also found that late hypoxia during early development suppressed the expression of muscle *igfbp3a* in individuals with enhanced size heterogeneity later in life^30^. Therefore, as previously stated, it is difficult to draw overarching conclusions on gene *igfbp* expression. However, a general trend for a number of muscle mRNA transcripts, including *igfbp5b*, *myod2*, *mef2c* and *fst,* was an offspring stimulated transcription by early hypoxic imprinting that was suppressed by late hypoxia. The precise mechanism remains elusive, though this finding is indicative of the existence of critical windows of development plasticity that respond differentially in fish facing the same or repeated environmental stimuli through early development. Meanwhile, the same pattern was found for *ghr1* in liver, which agrees with the observation that the induction of hepatic *ghr1* was beneficial under hypoxia in Atlantic salmon^59^. Hepatic *igfbp4* also emerged as an early hypoxic responsive gene, as reported for the hypoxic condition of glioma cells in other animal models^60^. The expression of hepatic *igfbp5* was also induced by hypoxia, but this up-regulation was limited to NNH or HNH fish when comparisons were made with normoxic control fish, which suggests some association with growth inhibition as reported in Atlantic salmon for chronically stressed fish^61^.

Changes in substrate preference and hypo-metabolic states are adaptive features across all the animal kingdom when individuals are facing predictable seasonal signals or unpredictable episodic stresses such as hypoxia, desiccation or traumatic surgical situations^62–64^. Besides, epigenetics allows pre-programming of offspring to high-altitude hypoxic environments by imprinting genes at the embryonic or placental interface, resulting in transgenerational and/or intra-generational heritable changes that affect gene expression^65^. Thus, adaptation of Tibetans and Quechuas from thousands of generations to high-altitude hypoxia is viewed as a down-regulation of maximum aerobic and anaerobic exercise capacities with a concomitant increase of the contribution of aerobic metabolism to whole energy supply^66^. However, in our experimental model, fish exposed to mild hypoxia during early life would share an increased basal metabolism with an enhanced contribution of anaerobic metabolism to whole energy supply. This was supported by the concurrence of a lowered circulating Hb concentration in association with increased circulating levels of lactate in HNN and HNH fish. Additionally, fish performance of HNN fish is not compromised later in life, whereas late or repeated hypoxic stimulus resulted in increased size heterogeneity several months later^30^. Also, history trajectories of O_2_ availability has a major impact on the composition of gut microbiota [Naya-Català et al*.*, unpublished results], with a now complex regulation and perhaps distal effects on productive traits other than more directly related to intestinal health^67,68^.

**Figure 3:**
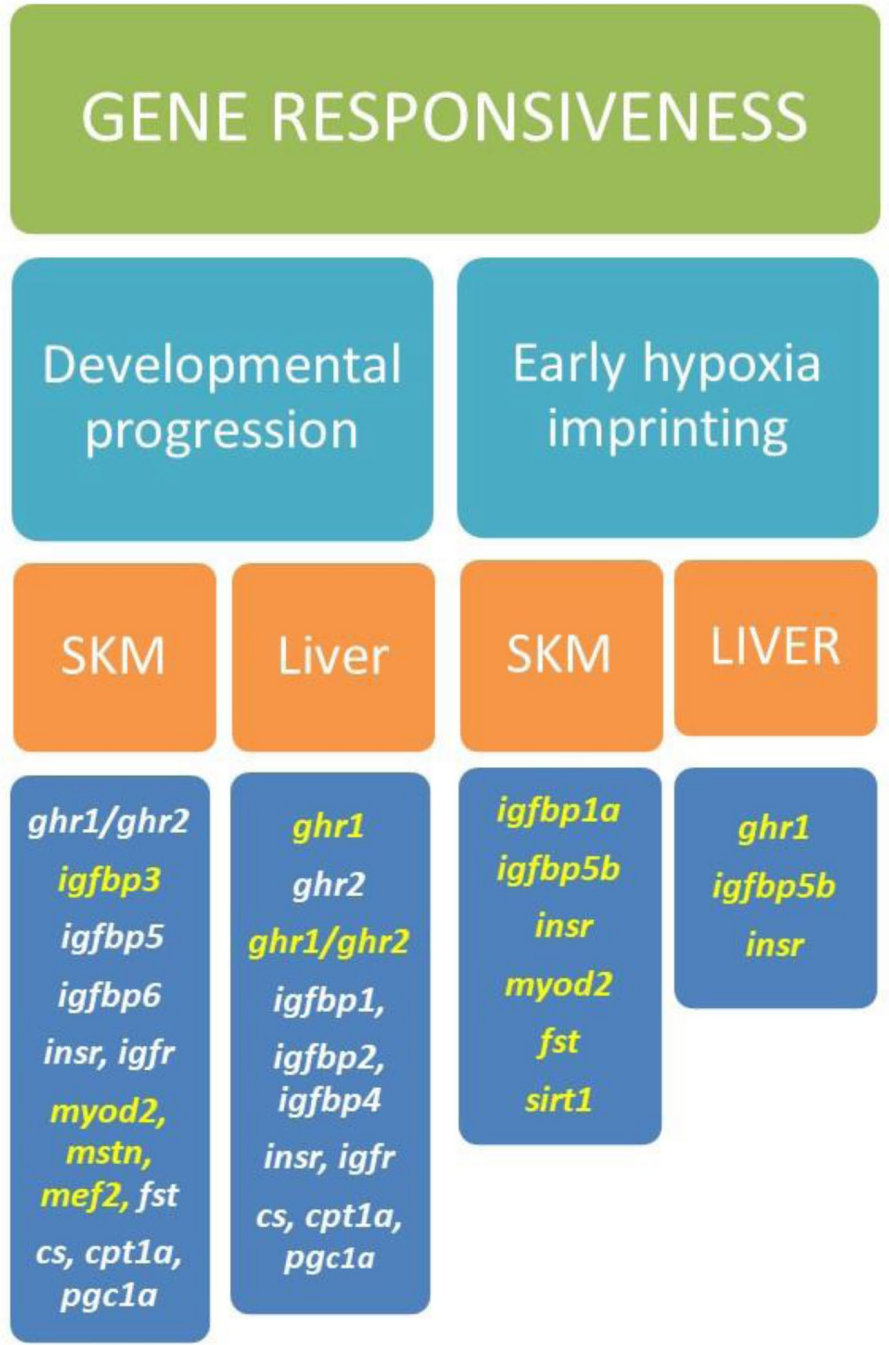
Schematic summary of progression of gilthead sea bream marker genes expression profiling in skeletal muscle and liver through development and as a response to early hypoxia. Genes with increased expression are highlighted in yellow, and those with decreased expression are highlighted in white.

In summary, the present study provides new insights in the regulation and function of Gh/Igf system in fish with especial emphasis on the effects of early hypoxia exposure. Such approach also contributed to understand the functional differentiation in a tissue-specific manner of fish duplicated paralogs of *ghrs*, *igfs* and *igfbps* (see Fig. 3). How this knowledge can contribute to establish robust criteria of larval and juvenile fish quality requires more research, though changes in the expression and the quotient expression ratio of duplicated *ghr/igf/igfbp* genes can serve as a measure of developmental progression and metabolic disturbances of the offspring.

## Methods

### Experimental setup

**Figure 4:**
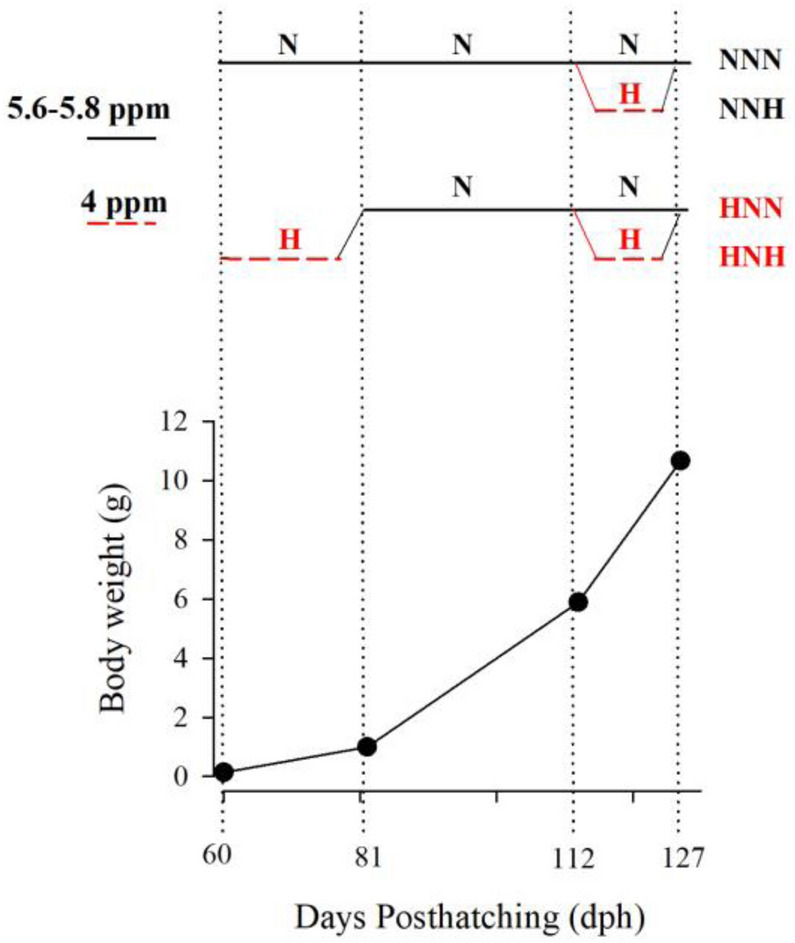
Experimental setup of hypoxia imprinting. Timing of normoxia (N) and hypoxia (H) exposure determines the four experimental groups: normoxic (NNN), early hypoxic (HNN), late hypoxic (NNH), and early and late hypoxic (HNH).

Gilthead sea bream larvae were transferred at 45 dph (May 2018) from the hatchery of Piscimar (Burriana, Castellón, Spain) to the facilities of IATS-CSIC. After an acclimation period of 15 days, 60 dph larvae (137 mg average weight) were distributed in six 90-L tanks (n = 150 larvae per tank) connected to two re-circulatory systems with control of water temperature (22–23 °C) and O_2_ concentration^28^. Feeding was carried out to visual satiety seven times per day with commercial pellets (0.2, 0.3, 0.5 mm Skretting Gemma Wean; 0.8 mm Skretting Perla Plus; 1.5 mm Biomar Intro Plus MT; 1.9, 3 mm Biomar EFICO YM 853). The daily percentage of provided feed along the experimental period (60–127 dph) ranged from 12 to 4%. Water quality was daily checked and O_2_ concentration was maintained high (85% saturation; 5.8 ppm) in normoxic tanks, whereas it was decreased gradually over the course of 24 h in fish exposed to mild hypoxia (50%; 3.6–3.8 ppm). The O_2_ concentration of the tanks was ramped to achieve the hypoxic condition according to the values of limiting oxygen saturation (LOS, defined as O_2_ levels where the maximal metabolic rates start to decrease with further reduction in dissolved O_2_) reported for this fish species at a given temperature^69^ This reduced O_2_ concentration was maintained for 21 days (60–81 dph) with the restoration of normoxia up to 112 dph. Then, fish from each tank were distributed in two additional tanks (40 fish per tank, 6.8–7.2 g average weight), and O_2_ levels remained high or low for 15 days. This resulted in four groups of fish according to their O_2_ availability history (NNN, normoxic; NNH, late mild hypoxic; HNN, early mild hypoxic; HNH, early and late mild hypoxic) (Fig. 4). After this last stage, overnight fasted fish were anesthetized with 100 mg/L 3-aminobenzoic acid ethyl ester (MS-222, Sigma, Saint Louis, MO, USA) for blood and tissue sampling. Blood was taken from caudal vessels with heparinized syringes. Prior to tissue collection, fish were killed by cervical section and representative portions of liver and white skeletal muscle were excised and immediately snap-frozen in liquid nitrogen and stored at − 80 °C until extraction of total RNA.

### Blood biochemistry

Hb was assessed using a Hemocue Hb 201 + (Hemocue, Sweden). Blood lactate was measured in deproteinized samples (perchloric acid 8%) by an enzymatic method based on the use of lactate oxidase and peroxidase (Spinreact S.A., Girona, Spain).

### RNA extraction and reverse transcription

Total RNA from whole larvae, liver and skeletal muscle at different sampling points was extracted using a MagMax-96 total RNA isolation kit (Life Technologies, Carlsbad, CA, USA). The RNA yield was > 3.5 µg with absorbance measures (A_260/280_) of 1.9–2.1. Synthesis of cDNA was performed with the High-Capacity cDNA Archive Kit (Applied Biosystems, Foster City, CA, USA), using random decamers and 500 ng of total RNA in a final volume of 100 µL. Reverse transcription (RT) reactions were incubated 10 min at 25 °C and 2 h at 37 °C, and negative control reactions were run without reverse transcriptase.

### Gene expression profiling

The 96-well PCR-array layout was designed for the simultaneous profiling of a panel of 28 genes, including markers of GH/IGF system (18), muscle cell growth and differentiation (5), and lipid and energy metabolism (5) (Table 1). qPCR reactions were performed using an iCycler IQ Real-time Detection System (Bio-Rad, Hercules, CA, USA). Diluted RT reactions were conveniently used for qPCR assays in a 25 µL volume in combination with a SYBR Green Master Mix (Bio-Rad, Hercules, CA, USA), and specific primers at a final concentration of 0.9 µM and an annealing temperature ranging between 58 and 62 °C (Supplemental Table 5). The program used for PCR amplification included an initial denaturation step at 95 °C for 3 min, followed by 40 cycles of denaturation for 15 s at 95 °C and annealing/extension for 60 s at 60 °C. All the pipetting operations were made by means of an EpMotion 5070 Liquid Handling Robot (Eppendorf, Hamburg, Germany) to improve data reproducibility. The efficiency of PCRs (> 92%) was checked, and the specificity of reactions was verified by analysis of melting curves (ramping rates of 0.5°C/10 s over a temperature range of 55–95 °C) and linearity of serial dilutions of RT reactions (> 0.97). Fluorescence data acquired during the extension phase were normalized by the delta-delta C_T_ method^70^ using β-actin as housekeeping gene due to its stability over time within each tissue (average C_T_ varied less than 0.3 within liver and muscle/whole larvae samples). For multi-gene analysis, data on gene expression were in reference to the expression level of insulin-like growth factor 2 receptor (*igf2r*) in liver, and insulin-like growth factor 1a receptor (*igf1ar)* in muscle and whole-larvae.

### Statistical analysis

Data on gene expression were analysed one-way analysis of variance (ANOVA) followed by the Student Newman–Keuls post-hoc test for comparisons among different groups. Paired comparisons with NNN group were conducted by *t *test. The significance level was set at *P* < 0.05. Analyses were performed using SigmaPlot v13 (Systat Software Inc, San Jose, CA, USA).

### Ethics declaration

All studies adhered to the ARRIVE Guidelines, were carried out in accordance with the guidelines of the European Union Council (2010/63/UE), and of the Spanish Government (RD 53/2013) for the use of animals in research, and were approved by the Ethics Committee of IATS-CSIC.

## Supplementary Information

Supplementary Tables.
