# Supplementary material for: Transcriptomic profiling of Gh/Igf system reveals a prompted tissue-specific differentiation and novel hypoxia responsive genes in gilthead sea bream
Source: Sci Rep. 2021 Aug 12;11:16466. doi: 10.1038/s41598-021-95408-6 (PMC8360970; doi:10.1038/s41598-021-95408-6)
Supplement: Supplementary file 1 — Supplementary Tables. [file 41598_2021_95408_MOESM1_ESM.pdf]

Supplemental Table 1. Gene expression profiling of whole body and skeletal muscle during early life stages. Data are the mean ± SEM of 6 fish. All values were in reference to the expression level of *igf2r* at 60 dph with an arbitrary assigned value of 1.

	WHOLE BODY 60dph	SKM 81dph	SKM 112dph	SKM 127dph	*P-value*
ghr1	1.04 ± 0.20	1.01 ± 0.07	1.02 ± 0.13	1.11 ± 0.08	0.905
ghr2	1.67 ± 0.25[a]	1.69 ± 0.10[a]	1.27 ± 0.31[ab]	0.65 ± 0.11[b]	0.009
igf1	0.89 ± 0.17[a]	0.64 ± 0.04[ab]	0.45 ± 0.08[bc]	0.20 ± 0.05[c]	<0.001
igf2	4.25 ± 0.74[a]	2.51 ± 0.21[b]	1.53 ± 0.15[c]	0.88 ± 0.13[c]	<0.001
igfbp1a	0.45 ± 0.04[a]	0.28 ± 0.03[b]	0.23 ± 0.02[b]	0.08 ± 0.01[c]	<0.001
igfbp1b	2.89 ± 1.12[a]	0.03 ± 0.02[b]	0.00 ± 0.00[b]	0.06 ± 0.05[b]	<0.001
igfbp2a	2.58 ± 0.62[a]	0.57 ± 0.08[b]	0.21 ± 0.04[b]	0.08 ± 0.05[b]	<0.001
igfbp2b	0.64 ± 0.21[a]	0.02 ± 0.02[b]	0.00 ± 0.00b	0.06 ± 0.04[b]	<0.001
igfbp3a	0.80 ± 0.06[c]	1.30 ± 0.09[bc]	1.83 ± 0.11[ab]	2.15 ± 0.24[a]	<0.001
igfbp3b	0.11 ± 0.02	0.12 ± 0.05	0.04 ± 0.01	0.01 ± 0.00	0.083
igfbp4	1.76 ± 0.40[a]	0.64 ± 0.05[b]	0.45 ± 0.09[bc]	0.10 ± 0.01[c]	<0.001
igfbp5a	1.76 ± 0.27[a]	1.37 ± 0.14[a]	0.90 ± 0.11[b]	0.17 ± 0.03[c]	<0.001
igfbp5b	4.26 ± 0.37[a]	2.97 ± 0.30[b]	2.40 ± 0.24[bc]	1.80 ± 0.07[c]	<0.001
igfbp6a	0.03 ± 0.00[ab]	0.06 ± 0.01[ab]	0.11 ± 0.04[a]	0.01 ± 0.00[b]	0.017
igfbp6b	0.43 ± 0.08[a]	0.40 ± 0.02[a]	0.34 ± 0.04[a]	0.11 ± 0.01[b]	<0.001
insr	1.57 ± 0.23[a]	1.05 ± 0.07[b]	0.89 ± 0.06[b]	0.54 ± 0.03[c]	<0.001
igf1ra	1.11 ± 0.13[a]	0.98 ± 0.06[ab]	0.73 ± 0.10[b]	0.34 ± 0.02[c]	<0.001
igf2r	1.02 ± 0.15[a]	0.32 ± 0.04[b]	0.27 ± 0.04[b]	0.16 ± 0.01[b]	<0.001
myod2	0.36 ± 0.11[c]	0.72 ± 0.06[bc]	0.98 ± 0.15[b]	1.52 ± 0.16[a]	<0.001
mstn/gdf8	0.16 ± 0.04[b]	0.42 ± 0.02[b]	0.46 ± 0.05[b]	1.08 ± 0.14[a]	<0.001
mef2a	4.26 ± 0.60[b]	7.00 ± 0.40[a]	7.38 ± 0.55[a]	7.13 ± 0.46[a]	0.007
mef2c	1.87 ± 0.31[b]	2.68 ± 0.12[a]	2.26 ± 0.13[ab]	2.60 ± 0.13[a]	0.015
fst	1.05 ± 0.11[a]	1.01 ± 0.06[a]	0.78 ± 0.10[a]	0.34 ± 0.05[b]	<0.001
cs	16.27 ± 1.88[a]	15.55 ± 1.14[a]	11.36 ± 1.30[ab]	10.42 ± 0.73[b]	0.007
cpt1a	4.96 ± 0.99[a]	3.38 ± 0.17[b]	2.15 ± 0.13[c]	2.26 ± 0.13[bc]	<0.001
pgc1α	0.85 ± 0.02[a]	0.39 ± 0.07[b]	0.24 ± 0.08[b]	0.15 ± 0.01[b]	<0.001
sirt1	0.43 ± 0.06[a]	0.25 ± 0.02[b]	0.20 ± 0.02[b]	0.10 ± 0.01[c]	<0.001
sirt2	0.70 ± 0.09[a]	0.40 ± 0.04[b]	0.34 ± 0.03[b]	0.26 ± 0.03[b]	<0.001

[1]*P-values* result from one-way ANOVA. Different superscript letters in each row indicate significant differences among sampling points (Student Newman-Keuls, $P < 0.05$).

Supplemental Table 2. Gene expression profiling of liver during early life stages. Data are the mean ± SEM of 6 fish. All values were in reference to the expression level of *igfr2* at 81 dph with an arbitrary assigned value of 1.

	LIVER 81dph	LIVER 112dph	LIVER 127dph	*P-value*
ghr1	5.49 ± 0.69[b]	9.25 ± 1.89[ab]	10.65 ± 0.97[a]	0.034
ghr2	13.92 ± 2.42[ab]	17.26 ± 1.21[a]	8.71 ± 0.84[b]	0.011
igf1	20.91 ± 3.83[b]	48.52 ± 9.77[a]	41.11 ± 4.44[ab]	0.017
igf2	24.79 ± 3.26	29.48 ± 2.39	23.04 ± 2.30	0.329
igfbp1a	0.48 ± 0.13[a]	0.22 ± 0.01[ab]	0.12 ± 0.02[b]	0.011
igfbp1b	59.57 ± 7.81[a]	24.85 ± 5.67[b]	6.20 ± 1.82[b]	<0.001
igfbp2a	28.77 ± 3.28[a]	33.58 ± 3.21[a]	10.45 ± 0.92[b]	<0.001
igfbp2b	16.48 ± 0.76[b]	22.02 ± 1.21[a]	11.44 ± 0.99[c]	<0.001
igfbp3a	0.05 ± 0.02	0.05 ± 0.01	0.03 ± 0.01	0.487
igfbp3b	0.23 ± 0.13	0.08 ± 0.01	0.03 ± 0.01	0.165
igfbp4	4.24 ± 0.52	4.91 ± 0.44	3.42 ± 0.39	0.096
igfbp5a	0.27 ± 0.03[a]	0.14 ± 0.02[b]	0.12 ± 0.01[b]	<0.001
igfbp5b	2.01 ± 0.18[a]	1.97 ± 0.14[a]	0.78 ± 0.07[b]	<0.001
igfbp6a	0.01 ± 0.00	0.01 ± 0.00	0.00 ± 0.00	0.052
igfbp6b	0.12 ± 0.04[a]	0.05 ± 0.01[ab]	0.03 ± 0.00[b]	0.031
insr	2.85 ± 0.23[b]	4.18 ± 0.24[a]	1.69 ± 0.11[c]	<0.001
Igf1ra	0.18 ± 0.02[b]	0.24 ± 0.02[a]	0.14 ± 0.01[b]	0.001
igfr2	1.01 ± 0.05[a]	0.94 ± 0.11[ab]	0.65 ± 0.08[b]	0.014
myod2	0.00 ± 0.00	0.00 ± 0.00	0.00 ± 0.00	1.000
mstn/gdf-8	0.00 ± 0.00	0.00 ± 0.00	0.00 ± 0.00	0.670
mef2a	2.50 ± 0.26[a]	2.89 ± 0.24[a]	1.71 ± 0.10[b]	0.005
mef2c	0.12 ± 0.01[a]	0.14 ± 0.01[a]	0.09 ± 0.01[b]	0.005
fst	0.06 ± 0.04	0.02 ± 0.01	0.01 ± 0.00	0.224
cs	5.47 ± 0.68[a]	5.07 ± 0.27[ab]	3.48 ± 0.22[b]	0.014
cpt1a	4.23 ± 0.54[a]	5.50 ± 0.38[a]	2.17 ± 0.23[b]	<0.001
pgc1α	1.21 ± 0.11[a]	1.50 ± 0.44[a]	0.23 ± 0.04[b]	0.010
sirt1	0.39 ± 0.04[a]	0.43 ± 0.02[a]	0.22 ± 0.02[b]	<0.001
sirt2	1.03 ± 0.12	1.31 ± 0.13	1.00 ± 0.11	0.167

[1]*P-values* result from one-way ANOVA. Different superscript letters in each row indicate significant differences among sampling points (Student Newman-Keuls, $P < 0.05$).

Supplemental Table 3. Gene expression profiling of white skeletal muscle of gilthead sea bream fingerlings (127 dph) with different history trajectories of O_2 availability. Data are the mean ± SEM of 6 fish. All values were in reference to the expression level of *igf1ra* of NNN fingerlings with an arbitrary assigned value of 1.

	NNN	HNN	NNH	HNH	P-value
ghr1	3.33 ± 0.25	3.98 ± 0.30	3.07 ± 0.40	3.69 ± 0.27	0.209
ghr2	1.97 ± 0.34	1.94 ± 0.23	1.75 ± 0.27	2.38 ± 0.29	0.516
igf1	0.62 ± 0.14	1.03 ± 0.18	0.70 ± 0.09	0.86 ± 0.16	0.262
igf2	2.65 ± 0.39	2.63 ± 0.22	2.57 ± 0.22	2.60 ± 0.23	0.997
igfbp1a	0.25 ± 0.02[b]	0.35 ± 0.03[ab]	0.43 ± 0.04[a]	0.43 ± 0.06[a]	0.022
igfbp1b	0.17 ± 0.15	0.02 ± 0.01	0.02 ± 0.01	0.07 ± 0.04	0.533
igfbp2a	0.24 ± 0.16	0.11 ± 0.02	0.13 ± 0.03	0.18 ± 0.08	0.785
igfbp2b	0.17 ± 0.13	0.06 ± 0.02	0.07 ± 0.02	0.15 ± 0.09	0.695
igfbp3a	6.47 ± 0.71[a]	6.22 ± 0.57[ab]	4.63 ± 0.57[ab]	4.30 ± 0.22[b]	0.022
igfbp3b	0.02 ± 0.01	0.03 ± 0.01	0.04 ± 0.01	0.03 ± 0.00	0.591
igfbp4	0.29 ± 0.04	0.40 ± 0.05	0.37 ± 0.10	0.36 ± 0.05	0.615
igfbp5a	0.51 ± 0.08	0.64 ± 0.09	0.78 ± 0.15	0.50 ± 0.08	0.222
igfbp5b	5.43 ± 0.20	7.26 ± 0.69	5.88 ± 0.42	5.63 ± 0.42	0.048
igfbp6a	0.03 ± 0.01	0.05 ± 0.01	0.06 ± 0.01	0.05 ± 0.02	0.314
igfbp6b	0.33 ± 0.03	0.41 ± 0.04	0.38 ± 0.05	0.28 ± 0.04	0.199
insr	1.61 ± 0.09[b]	2.29 ± 0.18[a]	2.03 ± 0.17[ab]	1.84 ± 0.06[ab]	0.014
igf1ra	1.01 ± 0.06	1.23 ± 0.08	1.21 ± 0.13	1.26 ± 0.10	0.260
igfr2	0.48 ± 0.03	0.64 ± 0.03	0.55 ± 0.07	0.53 ± 0.04	0.153
myod2	4.59 ± 0.47[b]	7.34 ± 0.65[a]	4.60 ± 0.22[b]	5.16 ± 0.33[b]	<0.001
mstn/gdf-8	3.25 ± 0.41	3.65 ± 0.46	4.73 ± 0.80	3.81 ± 0.31	0.301
mef2a	21.46 ± 1.39	27.50 ± 2.54	24.34 ± 2.02	23.59 ± 1.29	0.184
mef2c	7.81 ± 0.40[ab]	9.24 ± 0.46[a]	8.39 ± 0.39[ab]	7.68 ± 0.14[b]	0.029
fst	0.88 ± 0.09	1.43 ± 0.21	0.88 ± 0.12	0.96 ± 0.12	0.043
cs	31.38 ± 2.20	34.30 ± 2.64	33.40 ± 3.91	27.82 ± 0.78	0.337
cpt1a	6.81 ± 0.39	5.82 ± 0.26	5.17 ± 0.69	5.07 ± 0.42	0.057
pgc1α	0.46 ± 0.03	0.26 ± 0.03	0.42 ± 0.17	0.25 ± 0.07	0.303
sirt1	0.31 ± 0.03	0.41 ± 0.03	0.34 ± 0.02	0.35 ± 0.01	0.052

[1] *P-values* result from one-way ANOVA. Different superscript letters in each row indicate significant differences among experimental groups (Student Newman-Keuls $P < 0.05$).

Supplemental Table 4. Gene expression profiling of liver of gilthead sea bream fingerlings (127 dph) with different history trajectories of O_2 availability. Data are the mean ± SEM of 6 fish. All values are in reference to the expression level of *igfr2* of NNN fingerlings with an arbitrary assigned value of 1.

	**NNN**	**HNN**	**NNH**	**HNH**	***P-value***
ghr1	17.18 ± 1.56[ab]	24.47 ± 2.44[a]	14.21 ± 1.93[b]	14.18 ± 1.92[b]	0.005
ghr2	14.06 ± 1.36	14.08 ± 1.97	19.74 ± 3.13	21.07 ± 4.08	0.220
igf1	66.32 ± 7.16	68.68 ± 8.81	59.52 ± 8.56	80.67 ± 16.81	0.598
igf2	37.17 ± 3.72	35.35 ± 8.46	51.80 ± 6.46	53.24 ± 8.93	0.217
igfbp1a	0.19 ± 0.020	0.31 ± 0.03	0.22 ± 0.04	0.24 ± 0.06	0.185
igfbp1b	6.35 ± 2.17	5.75 ± 1.57	11.27 ± 1.72	10.51 ± 2.59	0.161
igfbp2a	16.87 ± 1.48	19.10 ± 1.28	16.94 ± 2.35	19.47 ± 3.74	0.805
igfbp2b	18.46 ± 1.59	22.29 ± 1.18	19.98 ± 3.19	21.58 ± 2.94	0.678
igfbp4	5.52 ± 0.64[b]	9.71 ± 0.83[a]	7.01 ± 0.47[ab]	8.05 ± 0.90[ab]	0.005
igfbp5b	1.26 ± 0.11[b]	1.57 ± 0.17[ab]	2.25 ± 0.40[ab]	2.34 ± 0.22[a]	0.017
insr	2.73 ± 0.17[b]	3.34 ± 0.18[ab]	3.73 ± 0.35[ab]	4.06 ± 0.47[a]	0.043
igf1ra	0.22 ± 0.01	0.26 ± 0.02	0.27 ± 0.02	0.29 ± 0.02	0.089
igf2r	1.04 ± 0.13	1.33 ± 0.11	1.58 ± 0.26	1.56 ± 0.21	0.179
cs	5.62 ± 0.36	5.53 ± 0.34	5.59 ± 0.64	5.27 ± 0.40	0.941
cpt1a	3.50 ± 0.38	2.69 ± 0.33	4.22 ± 0.36	4.86 ± 0.91	0.059
pgc1α	0.38 ± 0.06	0.33 ± 0.09	0.50 ± 0.11	0.39 ± 0.07	0.573
sirt1	0.36 ± 0.03	0.51 ± 0.04	0.50 ± 0.07	0.54 ± 0.05	0.062
sirt2	1.61 ± 0.18	1.87 ± 0.13	2.06 ± 0.30	1.95 ± 0.19	0.494

[1] *P-values* result from one-way ANOVA. Different superscript letters in each row indicate significant differences among dietary treatments (Student Newman-Keuls $P < 0.05$).

Supplemental Table 5. Primers for qPCR amplification.

Gene	Symbol	GenBank	Primer	Optimal T_a (ºC)	Efficiency (%)
Growth hormone receptor-type 1	*ghr1*	AF438176	F: ACC TGT CAG CCA CCA CAT GA<br>R: TCG TGC AGA TCT GGG TCG TA	59.6	92.36
Growth hormone receptor-type 2	*ghr2*	AY573601	F: GAG TGA ACC CGG CCT GAC AG<br>R: GCG GTG GTA TCT GAT TCA TGG T	59.2	96.41
Insulin-like growth factor 1	*igf1*	AY996779	F: TGT CTA GCG CTC TTT CCT TTC A<br>R: AGA GGG TGT GGC TAC AGG AGA TAC	58.1	98.77
Insulin-like growth factor 2	*igf2*	AY996778	F: TGG GAT CGT AGA GGA GTG TTG T<br>R: CTG TAG AGA GGT GGC CGA CA	60.4	94.12
Insulin-like growth factor binding protein 1a	*igfbp1a*	KM522771	F: ACA AAC CAA AAC AGT GCG AGT CCT C<br>R: CCG TTC CAA GAG TTC ACA CAC CAG	59.8	97.69
Insulin-like growth factor binding protein 1b	*igfbp1b*	MH577189	F: GCC AAA CAG TGT GAG TCA TC<br>R: ATC TTC TTC CCG TTC CAG G	60.1	99.10
Insulin-like growth factor binding protein 2a	*igfbp2a*	MH577190	F: CCA GCA AAG AGA CCA CCT<br>R: TCT TCA TCT CCT GCC TGT G	60.5	99.63
Insulin-like growth factor binding protein 2b	*igfpb2b*	AF377998	F: AGC GAT GTG TCC TGA GAT AGT GAG<br>R: GCA CCG TGG CGT GTA GAC C	62.5	95.09
Insulin-like growth factor binding protein 3a	*igfbp3a*	MH577191	F: ACA GGC GTG TGG AGT GTA<br>R: TGG TGC TGG CAG GTC AAG	60.8	98.92
Insulin-like growth factor binding protein 3b	*igfbp3b*	MH577192	F: GCC AGA TTA TGG TCC CTG TCG GAG AGA G<br>R: GTC TGT AAT CTT GAG GCT GCT GAG GAT GCT	60.6	93.05
Insulin-like growth factor binding protein 4	*igfbp4*	KM658998	F: GGC ATC AAA CAC CCG CAC AC<br>R: ATC CAC GCA CCA GCA CTT CC	58.2	97.78
Insulin-like growth factor binding protein 5a	*igfbp5a*	MH577193	F: GAA TCT CAC GAT GAC GCC AT<br>R: TGC TGA TGT GGT CTC TTC C	61.1	99.87
Insulin-like growth factor binding protein 5b	*igfpb5b*	MH577194	F: GCA AGC AGT GTA AGC CAT CTC<br>R: TGA ACG CCG TAC TTG TCC A	59.9	95.81
Insulin-like growth factor binding protein 6a	*igfbp6a*	MH577195	F: CGA CCC GAA TCA CGA CAT ATA CAT<br>R: ACT TGC CAC GCC GCT TAC	61.5	94.26

Insulin-like growth factor binding protein 6b	*igfbp6b*	MH577196	F: GAT TGC TCA CTG CGG ATC<br>R: GGA GGG ACA GAC CTT GAA	60.2	96.04
Insulin receptor	*insr*	KM522774	F: ACG GAC AGC AAG AAG GCA GAG AAT C<br>R: GGC TTC AAC GGT CGG ATC AGG T	60.2	93.89
Insulin-like growth factor 1a receptor	*igf1ra*	KJ591052	F: TCA ACG ACA AGT ACG ACT ACC GCT GCT<br>R: CAC ACT TTC TGG CAC TGG TTG GAG GTC	60.2	97.02
Insulin-like growth factor 2 receptor	*igf2r*	KM522776	F: ACA TTC GGG CAG CAC TCC TAA GAT<br>R: CCA GTT CAC CTC GTA GCG ACA GTT	59.8	94.92
Myogenic factor MYOD2	*myod2*	AF478569	F: CCA ACT GCT CTG ATG GCA TGA TGG ATT TC<br>R: GAC CGT TTG CTT CTC CTG GAC TCG TAT G	59.8	99.95
Myostatin/Growth differentiation factor 8	*mstn/ gdf8*	AF258448	F: AAG AGC AGA TCA TCT ACG GCA AGA TCC<br>R: TCA AGA GCA TCC ACA ACG GTC TAC CA	59.8	96.37
Myocyte-specific enhancer factor 2A	*mef2a*	KM522777	F: ATG GAC GAG AGG AAC AGG CAG GTT A<br>R: GGC TAT CTC ACA GTC ACA TAG TAC GCT CAG	61.1	96.59
Myocyte-specific enhancer factor 2C	*mef2c*	KM522778	F: TAG CAA CTC CCA CTC TAC CAG GAC AAG<br>R: GGA ATA CTC GGC ACC ATA AGA AGT CG	60.8	92.04
Follistatin	*fst*	AY544167	F: GGA CCA GAC AAA CAA CGC ATA TTG<br>R: CAT AGA TGA TCC CGT CGT TTC CAC	60.9	96.28
Citrate synthase	*cs*	JX975229	F: TCC AGG AGG TGA CGA GCC<br>R: GTG ACC AGC AGC CAG AAG AG	59.9	95.33
Carnitine palmitoyltransferase 1a	*cpt1a*	JQ308822	F: GTG CCT TCG TTC GTT CCA TGA TC<br>R: TGA TGC TTA TCT GCT GCC TGT TTG	59.1	92.94
Proliferator-activated receptor gamma coactivator 1 alpha	*pgc1α*	JX975264	F: CGT GGG ACA GGT GTA ACC AGG ACT C<br>R: ACC AAC CAA GGC AGC ACA CTC TAA TTC T	60.3	95.97
Sirtuin 1	*sirt1*	KF018666	F: GGT TCC TAC AGT TTC ATC CAG CAG CAC ATC<br>R: CCT CAG AAT GGT CCT CGG ATC GGT CTC	59.9	95.42
Sirtuin 2	*sirt2*	KF018667	F: GAA CAA TCC GAC GAC AGC AGT GAA G<br>R: AGG TTA CGC AGG AAG TCC ATC TCT	60.0	97.75
β-actin	*actb*	KY388508	F: TCC TGC GGA ATC CAT GAG A<br>R: GAC GTC GCA CTT CAT GAT GCT	58.9	94.61
